# Scalp Acupuncture for Acute Ischemic Stroke: A Meta-Analysis of Randomized Controlled Trials

**DOI:** 10.1155/2012/480950

**Published:** 2012-12-04

**Authors:** Yan Wang, Jiangang Shen, Xiu-min Wang, Deng-lei Fu, Chao-yang Chen, Ling-yan Lu, Lin Lu, Cheng-long Xie, Jian-qiao Fang, Guo-qing Zheng

**Affiliations:** ^1^The Center of Neurology and Rehabilitation, The Second Affiliated Hospital of Wenzhou Medical College, Wenzhou 325027, China; ^2^School of Chinese Medicine, University of Hong Kong, Hong Kong, China; ^3^The Second People's Hospital of Hefei City, The Affiliated Hefei Hospital of Anhui Medical University, Hefei 230022, China; ^4^Department of Neurobiology Acupuncture Research, Zhejiang Chinese Medical University, Hangzhou 310005, China

## Abstract

Scalp acupuncture (SA) is a commonly used therapeutic approach for stroke throughout China and elsewhere in the world. The objective of this study was to assess clinical efficacy and safety of SA for acute ischemic stroke. A systematical literature search of 6 databases was conducted to identify randomized controlled trials (RCTs) of SA for acute ischemic stroke compared with western conventional medicines (WCMs). All statistical analyses were performed by the Rev Man Version 5.0. Eight studies with 538 participants were included in the studies. The studies were deemed to have an unclear risk of bias based on the Cochrane Back Review Group. Compared with the WCM, 6 RCTs showed significant effects of SA for improving neurological deficit scores (*P* < 0.01); 4 RCTs showed significant effects of SA for favoring the clinical effective rate (*P* < 0.01) However, the adverse events have not been documented. In conclusion, SA appears to be able to improve neurological deficit score and the clinical effective rate when compared with WCM, though the beneficial effect from SA is possibly overvalued because of generally low methodology of the included trials. No evidence is available for adverse effects. Rigorous well-designed clinical trials are needed.

## 1. Introduction

Stroke is the second most common cause of death preceded only by heart attacks and the major cause of disability in the Western world [1] and may soon become the leading cause of death worldwide [2]. More than two thirds of the burden of global stroke occurs in developing (low- and middle-income) countries [3]. Ischemic stroke is the most common subtype of stroke, accounting for about 80% of all strokes. Moreover, the epidemiological data showed that from 1984 to 2004 the incidence of ischemic stroke increased by 8.7% though the incidence of hemorrhagic stroke decreased by 1.7% every year in China [4]. Therefore, prevention and treatment of stroke, especially ischemic stroke in China has become an even greater challenge than that in the developed countries. However, the optimization of modern clinical treatment with acute ischemic stroke was only an integrated and systematic approach with thrombolysis, if indicated, and aggressive supportive care [5]. Thrombolysis agents, such as recombinant tissue plasminogen activator (rt-PA), were indicated for ischemic stroke if administered within 4.5 hours of stroke onset. However, bleeding risk, mortality rate (evident at 7 to 10 days and at final followup), and the incidence of intracranial haemorrhages have been reported to increase with the use of thrombolytic agents [6]. Thus, complementary and alternative medicine (CAM) therapies are increasingly used in patients with stroke adjunct to conventional treatment.

The most appreciable distinction between China and the West in treating stroke is the application of traditional Chinese medicine (TCM) which includes herbal medicine, acupuncture, and other nonmedication therapies [7, 8]. Acupuncture has been used in many diseases, especially in stroke victims, as a medical modality for over 3000 years [9]. In modern time, at least 9 systematic reviews and meta-analysis have been conducted in the effectiveness and safety of acupuncture for stroke [10]. Among of them, 1 focused on acute stroke; 1 focused on subacute and chronic stroke; 5 observed the variable interval after stroke onset; and 2 addressed dysphagia after stroke. However, the results regarding the effectiveness of acupuncture for stroke recovery were inconclusive. Especially, the review from Kong and colleagues [11] included only randomized clinical trials that compared acupuncture with sham acupuncture regardless of the stages (acute, subacute, or chronic), types of stroke (ischemic or hemorrhagic), and the number and regimen of acupoints selected, and the results did not show a positive effect of acupuncture as a treatment for functional recovery after stroke. 

Scalp acupuncture (SA) is one of the several specialized acupuncture techniques with a specific body location, in which a filiform needle is used to penetrate specific stimulation areas on the scalp. Although SA has been practiced for thousands of years, SA develops very fast only in recent decades [12]. During the 1970s, SA was developed as a complete microacupuncture system based on the traditional acupuncture science, modern anatomy, neurophysiology, and bioholographic theory [12]. In 1984, a standard of nomenclature for acupuncture points was developed and reconfirmed in 1989, indicating 14 therapeutic lines or zones based on a combination of the thoughts of the different schools of scalp acupuncture [12]. *A Proposed Standard International Acupuncture Nomenclature: *3.6 Scalp acupuncture lines was formally published in 1991 by WHO [13]. In modern time, SA is often applied for treatment of central nervous system disorders and various acute or chronic pains and has achieved specific curative effects [14]. SA therapy for both ischemic stroke and hemorrhagic stroke has been empirically established and widely used in clinics in China [15, 16] and elsewhere in the world [17]. Although SA is commonly used in the acute, recovery, and sequelae stages of patients with ischemic stroke, the most important period of recovery is at the acute and subacute stage during the clinical course of ischemic stroke [18]. Therefore, the objective of present systematic review was to assess clinical efficacy and safety of SA for acute ischemic stroke.

## 2. Methods

The PRISMA (Preferred Reporting Items for Systematic Reviews and Meta-Analyses) Statement guidelines was followed during all stages of the design, implementation, and reporting of this meta-analysis [19].

### 2.1. Eligibility Criteria

Only true randomised controlled trials (RCTs) for evaluating the effects of SA therapy on acute ischemic stroke were included, regardless of blinding, publication status or language. Quasi-RCTs were not considered such as using the admission sequence for treatment allocation.

Participants with any age or sex and within 14 days of onset of acute ischemic stroke were eligible. The diagnostic criteria were adopted in accordance with national criteria* Diagnostic Essentials of Various Cerebrovascular Diseases* in 1986 [20] or the revised version national criteria in 1995 [21]. Diagnosis of stroke had to be confirmed with CT/MRI scan and haemorrhagic stroke excluded.

Studies were included if the intervention was SA therapy, regardless of the sources or methods of stimulation (e.g., scalp penetration acupuncture or electrical stimulation), the times of treatment, or the length of treatment period. Acupoint selection was limited to the head; studies using ear acupoints were excluded. The patients at the trial group were given SA therapy plus the western conventional medication (WCM) which was similar to the control group. WCM refers to the combination of needed therapies of the following aspects: (1) general supportive care mainly includes (A) airway, ventilatory support and supplemental oxygen, (B) cardiac monitoring and treatment, (C) temperature, (D) blood pressure, (E) blood sugar, and (F) nutrition; (2) specialized care mainly include a variety of measures to improve cerebral blood circulation (such as antiplatelet agents, anticoagulants, fibrinogen-depleting agents, volume expansion, and vasodilators, except thrombolytic agents) and neuroprotective agents; (3) treatment of acute complications mainly includes (A) brain edema and elevated intracranial pressure, (B) seizures, (C) dysphagia, (D) pneumonia, (E) voiding dysfunction and urinary tract infections, and (F) deep vein thrombosis. The intervention for control group included only WCM treatments. Studies comparing SA therapy to another form of acupuncture or combining SA and acupuncture or Chinese herbal medicine were excluded.

The outcome measures were neurological deficit score, the clinical effective rate and adverse events at the end of the treatment course. The neurological deficit score was adopted based on the Modified Edinburgh-Scandinavian Stroke Scale, which was recommended at the Second and revised at the Fourth National Cerebrovascular Diseases Conference in China in 1995 [21].

### 2.2. Information Sources and Search Strategy

Medical literature retrieval was performed by 2 investigators (WY and WXM) in the following databases: CENTRAL (The Cochrane Library 2012, Issue 1), PubMed (1950-December 2011), EMBASE (1980–2011), Chinese Biomedical Database (1978–2011), Wanfang Database (1998–2011), and China Hospital Knowledge Database (1979–2011). We used the combining text terms and, where appropriate, MeSH terms for scalp acupuncture (“scalp acupuncture” or “head acupuncture” or “cranial acupuncture” or “cephalic acupuncture” or “scalp electric acupuncture” or “scalp penetration acupuncture”) and stroke (“cerebrovascular disorders” or “cerebrovascular disease” or “stroke” or “brain infarction” or “apoplexy”). The search terms were combined with the “explode” feature. No limits were applied for language and country. 

### 2.3. Study Selection and Data Collection Process

Two investigators (WY and WXM) screened the titles and abstracts to select potential references in an unblinded standardized manner. Full articles for all potentially relevant studies were retrieved. The 2 investigators then read the selected papers independently and made a final selective decision. Disagreements were settled through discussion or consultation with a third author (ZGQ). 

Two investigators (WY and WXM) independently collected data on study characteristics, including patients, methods, interventions, and outcomes, into a standardized data extraction form for eligible studies. Reasons for the exclusion of studies were recorded. Disagreements were settled by discussion and consensus with a third member (ZGQ). 

### 2.4. Risk of Bias in Individual Studies

Two investigators independently (WY and WXM) assessed risk of bias for each included article, using the twelve criteria recommended by the Cochrane Back Review Group [30]. Disagreements were resolved through discussion with a third author (ZGQ).

### 2.5. Synthesis of Results

Heterogeneity between trial results was tested using a standard chi-square test, and we also calculated the *I* ^2^ statistic. For continuous outcomes, weighted mean difference (WMD) was calculated. For dichotomous outcomes, relative risk (RR) and 95% confidence intervals was calculated. Publication bias was assessed graphically using a funnel plot. All statistical analyses were performed by the Rev Man Version 5.0.

## 3. Results

### 3.1. Study Selection

**Figure 1:**
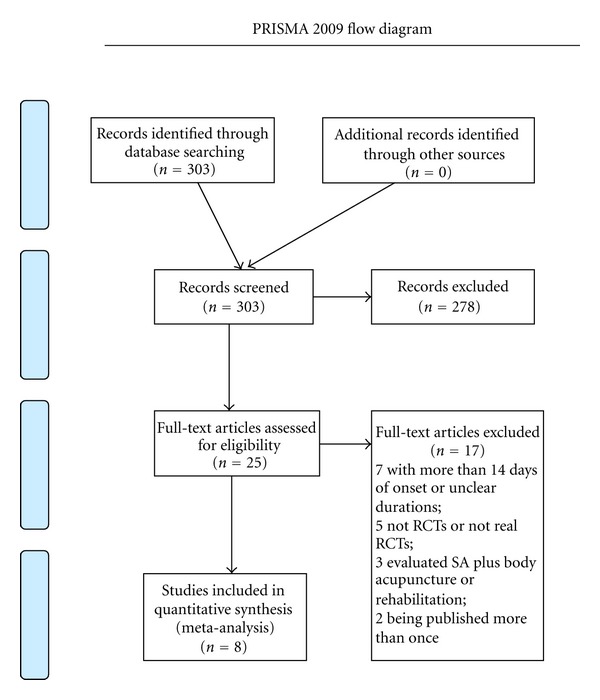
Flow diagram for literature search.

We identified and selected 303 papers by titles and abstracts. Among of them, 278 papers were excluded because they were case reports, lacked a comparison group, were not reports of clinical trials, or studies not focused on the scalp acupuncture treatment of acute ischemic stroke. Of the remaining 25 articles, 7 articles were excluded because they reported a sub-acute ischemic stroke with more than 14 days of onset or unclear durations; 5 articles were not RCTs or not real RCTs such as using admission sequence for treatment allocation; 3 articles evaluated SA plus body acupuncture or rehabilitation; 2 studies were suspected of being published more than once by the authors or publishers. We attempted but failed finally to contact the author for further information about the allocation process. Eventually, 8 eligible studies [22–29] were identified and included in this meta-analysis. The screening process is summarized in a flow diagram (Figure 1).

### 3.2. Study Characteristics

The 8 studies were all conducted by Chinese investigators and published between 1996 and 2011. Each study was performed in a single center. Five hundred and thirty-eight participants of Chinese ethnicity were included in the 8 studies, of whom 302 were male and 236 were female. The participants ranged in age from 38 to 75 years old, while one study did not mention the age range of participants [27]. One study was three-group design study [28], in which both bilateral scalp penetration needling and ipsilateral (disease-side) scalp penetration needling were compared with the WCM control. The national diagnostic criterion in 1995 was used in 7 studies; while the national diagnostic criterion in 1986 was used in 1 study [22]. The period of treatment varied from 10 days to 8 weeks. Clinical effective rate was observed in 4 studies and neurological deficit score in 6 studies. Detailed characteristics of included studies are listed in Table 1.

### 3.3. Results of Individual Studies

The twelve criteria recommended by the Cochrane Back Review Group were used to assess the risk of bias of each study. The number of criteria complied varied from 4/12 to 7/12. All of the studies included suggested randomization, but only 2 studies reported the method of random sequences generation [26, 27]. No study referred to allocation concealment, and blinding procedures. 2 studies reported intention-to-treat analyses and drop-out data [24, 26]. All the studies presented selective reporting, characterized similarity of baseline and reported the co-intervention. Adequate and acceptable compliance seemed to have been described in all of the included studies. Timing of outcome assessments in all studies was similar. In general, all 8 RCTs were deemed to have an unclear risk of bias based on the Cochrane Risk of Bias tool. The methodological quality of each study is described in Table 2. 

### 3.4. Synthesis of Results

#### 3.4.1. Neurological Deficit Scores

**Figure 2:**
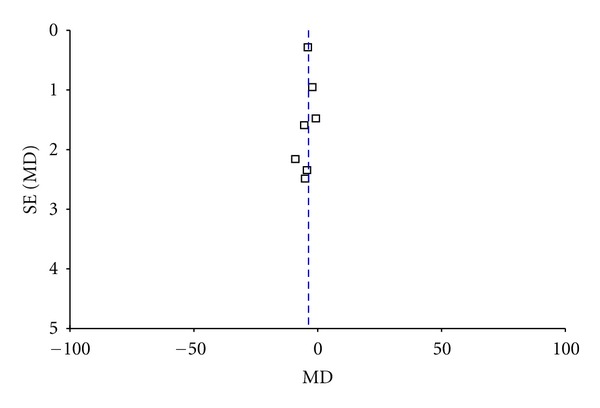
Funnel plot of comparison: scalp acupuncture versus western conventional medicines: neurologic deficit scores.

Data extracted from 6 studies showed heterogeneity in the consistency of the trial results (heterogeneity: chi-square = 14.02, *P* = 0.03, *I* ^2^ = 77%). Thus, the random-effects model should be employed for statistical analysis. The combined effects of 6 independent trial results showed that SA therapy had further improved the neurological deficit scores in patients with acute ischemic stroke when compared with WCM control (*n* = 223, WMD: −3.89, 95% CI: −5.36 to −2.43, *z* = 5.22, *P* < 0.00001), Table 3. The funnel plot was asymmetric. There exists a publication bias in the 6 independent trials (Figure 2).

#### 3.4.2. The Clinical Effective Rate

Data extracted from 4 studies showed homogeneity in the consistency of the trial results (heterogeneity: chi-square = 0.57, *P* = 0.90; *I* ^2^ = 0%). Thus, the fixed-effects model should be employed for statistical analysis. The combined effects of 4 independent trial results showed that SA therapy had further improved the clinical effective rate in patients with acute ischemic stroke when compared with WCM control (*n* = 153, RR = 1.23, 95% CI, 1.11–1.37, *z* = 3.85, *P* < 0.01), Table 4.

#### 3.4.3. Adverse Events

None of the trials reported adverse effects.

## 4. Discussion

### 4.1. Summary of Evidence

To our knowledge, this is the first meta-analysis of SA therapy in the treatment of acute ischemic stroke to date. 8 studies with 538 individuals suffering from acute ischemic stroke were selected for present meta-analysis. The main findings were that SA therapy can further improve neurological deficit score and the clinical effective rate in patients with acute ischemic stroke when compared with WCM control. However, the evidence is insufficient to warrant a clinical recommendation due to the generally low methodological quality of the included studies. In addition, there is no evidence available on safety because none of the trials reported adverse effects.

### 4.2. Limitations

In September 2004, the members of the International Committee of Medical Journal Editors (ICMJE) published a statement requiring that all clinical trials must be registered in order to be considered for publication [31]. Clinical trial registration will improve research transparency and will ultimately strengthen the validity and value of the scientific evidence base. However, none of included studies had been registered.

There are several methodological limitations in the primary studies. The included trials were of generally poor methodological quality with regard to the method of sample calculation, randomization, allocation concealment and blinding of assessment. Moreover, no eligible sham SA-controlled trials have been included and this may lead to an increase in the risk of performance bias. Lack of blinding in randomized trials has been shown to be associated with more exaggerated odds ratios by 9% on average for intervention effect [32].

The revised standards for reporting interventions in clinical trials of acupuncture (STRICTA) [33] include 6 items and 17 subitems, which set the reporting guidelines for the acupuncture rationale, the details of needling, the treatment regimen, other components of treatment, the practitioner background, and the control or comparator intervention. However, there were no sufficient reports on these items in the included trials, especially for acupuncture techniques such as the number of acupoints, the depth of insertion, responses elicited, needle stimulation, and needle type, and no trial mentioned practitioners' background. A misleading result could be exhibited if the treatment schedules were inadequate or administered by unskilled practitioners. 

The clinical heterogeneity would be very significant due to the variations. Only 2 trials were conducted based on the *A Proposed Standard International Acupuncture Nomenclature* by WHO. 6 studies applied electrical stimulation with variable frequency, while the other 2 studies used manual stimulation of acupuncture needles. The difference between the effects of electroacupuncture and manual acupuncture could not be determined. The available data from the included trials were only secondary outcomes, and none used primary outcomes such as death or dependency for comparison. Duncan et al. [34] recommended that acute stroke trials should include extended/instrumental activities and advanced mobility as components of the primary outcome measure, with outcome assessment being undertaken at 6 months. Therefore, using primary outcome such as death or dependency will provide useful data for this therapy. In addition, the clinical efficacy, which was classified as cure, markedly effective, effective, and ineffective, is not internationally recognized, and it is not accurate for the assessment of the effect. 

Researchers participating in a clinical trial must report all adverse events. However, none of the included trials reported whether any adverse events relevant to SA were apparent in patients with acute ischemic stroke. 

The funnel plot asymmetry suggests the possibility of publication bias. In addition, language bias may exist because all included trials were published in Chinese. Vickers and colleagues [35] indicated that some Asian countries including China publish unusually high proportions of positive results. Although great efforts were made to retrieve all trials on the subject, we still could not exclude the possibility that studies with negative findings remain unpublished.

### 4.3. Implication for Practice

From the evidence available in the present systematic review, there is no conclusive evidence for the routine use of SA for acute ischemic stroke. Thus, the continuing and increasing limited evidence to support the application of SA is noteworthy. However, SA appears to be able to improve neurological deficit score and the clinical effective rate when compared with WCM control. With the widespread use of SA, we suggested the onus being on clinicians to provide the clear evidence for its use.

Acupuncture appears to be a safe treatment when used in the acute phase of stroke, with severe adverse events occurring very rarely [36]. A NIH consensus report also stated that one of the advantages of acupuncture was that the incidence of adverse effects is substantially lower than that of many other accepted medical interventions [37]. However, various types of acupuncture-related adverse events have been reported in China [38] and other countries [39]. Owing to most of acupuncture-related adverse events as a result of inappropriate technique [38], SA may be also considered inherently safe in the hands of well-trained practitioners. Unfortunately, no evidence is available from present studies to support or refute the safety of SA.

### 4.4. Implication for Research

Further trials should improve in the methodological quality of RCTs as follows: (1) all clinical trials registered according to ICMJE statement [31]; (2) sample size estimated by statistical calculation; (3) reporting of the adequate generation of the allocation sequence and adequate allocation concealment; (4) a clear description of the blinding; (5) use of sham SA-control; (6) a clear definition of the modality of SA, especially the international standard of SA lines by WHO; (7) the skilled practitioners should be selected to perform the SA manipulation and keep the consistency for all the time, and select suitable number of treatment sessions and frequency of treatment; (8) the balance of basic demographic data and baseline disease staging; (9) use of standard and validated outcome measures, and reporting important clinical outcome measures such as death, dependency, and quality of life for at least 6-month followup. Psychometric properties include validity, reliability, and sensitivity to change. An assessment tool should be scientifically sound in terms of three basic psychometric properties: reliability, validity, and responsiveness [40]. Thus, using standardised outcome measures with acceptable psychometric properties is needed in future studies; (10) SA-related adverse events should be rigorously assessed by standardized monitoring and an effective self-report system; and (11) the trials must be reported by using Revised standards for reporting interventions in clinical trials of acupuncture (STRICTA) [33] and some additional key elements in the reporting about acupuncture trials [41].

## 5. Summary

Based on the results of present systematic review, SA is significantly effective in improving neurological deficit score and clinical effective rate when compared with WCM control, though the beneficial effect from SA is possibly overvalued because of generally low methodology of the included trials. No adverse events were documented in these RCTs studies. More trials should be thoroughly focused on the experimental design and monitoring of adverse events. 

## Figures and Tables

**Table 1:** Characteristics of the included studies.

Included trials	Eligibility criteria1	Study designs	Gender (male/female); age (years)		Course of disease	Interventions (*n*) Drug/dosage		Course of treatment	Outcomes	Intergroup differences
			Trial	Control		Trial	Control			
Tang and Sun 1996 [22]	CMADS in 1986	RCT	*n* = 63 (30 : 33), Average of 64.2	41/22;	<7 d	SA#	WCM##	15 d	(1) clinical efficacy (2) Limb power recovery (3) Level of rehabilitation (4) SOD, MDA, PGI2, TAX2 (5) SEP	(1) *P* < 0.05 (2) *P* < 0.05 (3) *P* < 0.05 (4) *P* < 0.05 (5) *P* < 0.05

Yu et al. 2004 [23]	CMADS in 1995	RCT	19/11; 45–74	18/12; 46–74	<7 d	SA#	WCM##	10 d	(1) NDS (2) ADL (BI) (3) Blood MDA content	(1) *P* < 0.05 (2) *P* < 0.05 (3) *P* < 0.05

Wei et al. 2005 [24]	CMADS in 1995	RCT	20/16; Average of 59.44	22/14; Average of 62.26	Average of 5.1 d	SA#	WCM##	8 w	(1) clinical efficacy (2) NDS (3) ADL (FIM) (4) SIAS score	(1) *P* < 0.05 (2) *P* < 0.05 (3) *P* < 0.05 (4) *P* < 0.05

Xu et al. 2007 [25]	CMADS in 1995	RCT	33/28; Average of 62.33	29/32; Average of 59.83	6 h–3 d	SA#	WCM##	14 d	(1) clinical efficacy (2) NDS	(1) *P* < 0.05 (2) *P* < 0.05

Chen and Jing 2008 [26]	CMADS in 1995	RCT (statistical software)	18/14; —	14/16; —	<7 d	SA#	WCM##	2 w	(1) clinical efficacy (2) Kubota's water drinking test	(1) *P* < 0.01 (2) *P* < 0.01

Yu et al. 2010 [27]	CMADS in 1995	RCT (random number form)	*n* = 40 (20 : 20), 45–75 y	26/14;	<7 d	SA#	WCM##	10 d	(1) NDS (2) TCM symptom accumulated points (3) SOD activity (4) MDA content	(1) *P* < 0.05 (2) *P* < 0.05 (3) *P* < 0.05 (4) *P* < 0.05

Zhang et al. 2011b* [28]	CMADS in 1995	RCT	A group: 14/9; 59.8 ± 7.1	12/11; 58.0 ± 6.8	24 h	SA#	WCM##	30 d	(1) NDS (2) VEGF (test at 10th d)	(1) *P* < 0.05 (2) *P* < 0.05

Zhang et al. 2011a* [28]			B group: 13/10; 56.5 ± 6.5				WCM ##			(1) *P* < 0.05 (2) *P* < 0.05

Zhu and Huang 2011 [29]	CMADS in 1995	RCT	9/11; 59.07 ± 11.49	14/6; 56.03 ± 8.70	<7 d	SA#	WCM##	30 d	(1) NDS (2) Ca^2+^ content in serum	(1) *P* < 0.05 (2) *P* < 0.05

ADL: Activity of Daily Living Scale; BI: Bathel Index; CMADS: Chinese Medical Association Diagnosis Standard; d: day(s); FIM: Functional Independence Measure; h: hour(s); FMA: Fugl-Meyer Motor Assessment; m: months; MDA: Malondialdehyde; NDS: neurological deficit score; PGI2: prostacyclin; RCT: randomized controlled trial; SA: scalp acupuncture; SEP: Somatosensory Evoked Potentials; SIAS: Stroke Impairment Assessment Set; SOD: Superoxide Dismutase; TAX2: thromboxane A2; TCM: Traditional Chinese Medicine; VEGF: Vascular endothelial growth factor; w: week(s); WCM: western conventional medication; #: the same as the control group. WCM## refer to the combination of needed therapies of the following aspects: (1) general supportive care mainly includes (A) airway, ventilatory support and supplemental oxygen, (B) cardiac monitoring and treatment, (C) temperature, (D) blood pressure, (E) blood sugar, and (F) nutrition; (2) specialized care mainly include a variety of measures to improve cerebral blood circulation (such as antiplatelet agents, anticoagulants, fibrinogen-depleting agents, volume expansion, and vasodilators, except thrombolytic agents) and neuroprotective agents; (3) treatment of acute complications mainly includes (A) brain edema and elevated intracranial pressure, (B) seizures, (C) dysphagia, (D) pneumonia, (E)voiding dysfunction and urinary tract infections, and (F) deep vein thrombosis. *A group: bilateral scalp penetration needling; B group: ipsilateral (disease-side) scalp penetration needling.

**Table 2:** The included trials scored according to the risk of bias criteria.

	A	B	C	D	E	F	G	H	I	J	K	L
Tang and Sun 1996 [22]	−	−	−	−	−	?	+	?	+	+	−	+
Yu et al. 2004 [23]	−	−	−	−	−	+	+	?	+	+	+	+
Wei et al. 2005 [24]	−	−	−	−	−	−	−	?	+	+	+	−
Xu et al. 2007 [25]	−	−	−	−	−	+	+	?	+	+	+	+
Chen and Jing 2008 [26]	+	−	−	−	−	−	+	?	+	+	+	+
Yu et al. 2010 [27]	+	−	−	−	−	+	+	?	+	+	+	+
Zhang et al. 2011 [28]	−	−	−	−	−	+	+	?	+	+	+	+
Zhu and Huang 2011 [29]	−	−	−	**−**	−	+	+	?	+	+	+	+

A: adequate sequence generation; B: concealment of allocation; C: Blinding (patient); D: blinding (investigator); E: blinding (assessor); F: intention-to-treat analysis (ITT analysis); G: incomplete outcome data addressed(drop-outs); H: free of selective reporting; I: similarity at baseline; J: cointerventions constant; K: compliance acceptable; L: timing outcome assessments similar. +: Yes, −: No, ?: Unclear.

**Table 3:** Forest plot of comparison: scalp acupuncture versus western conventional medicines: neurologic deficit scores.

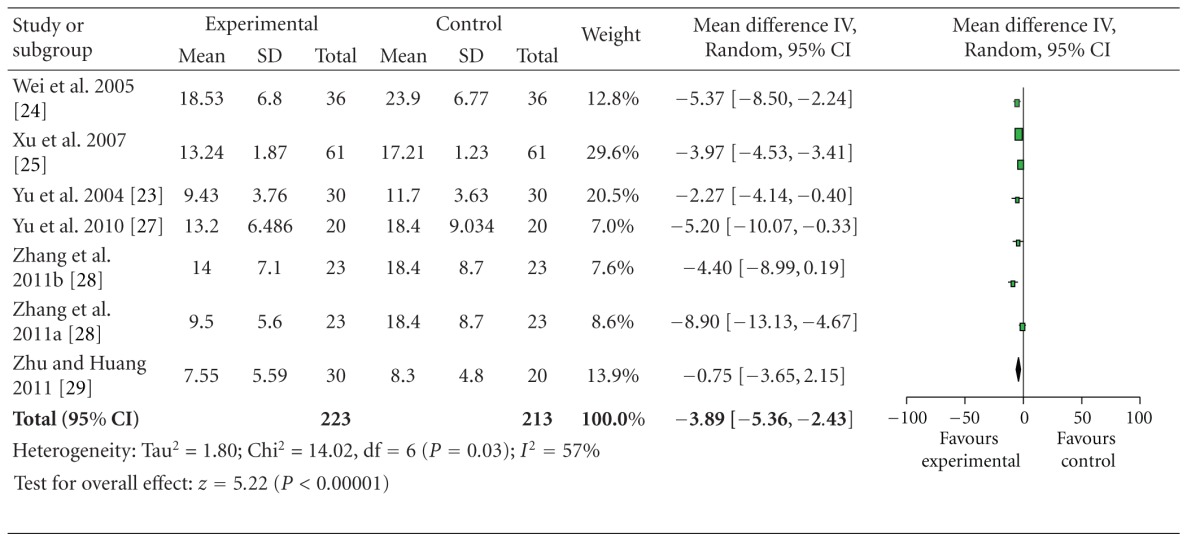

**Table 4:** Forest plot of comparison: scalp acupuncture versus western conventional medicines: the clinical effective rate.

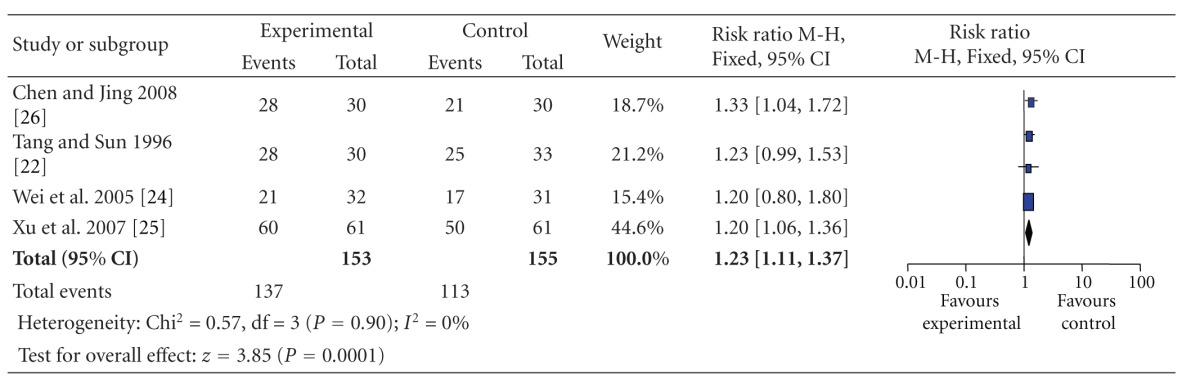

## References

[1] Donnan GA, Fisher M, Macleod M, Davis SM (2008). Stroke. *The Lancet*.

[2] Feigin VL (2005). Stroke epidemiology in the developing world. *The Lancet*.

[3] Feigin VL (2007). Stroke in developing countries: can the epidemic be stopped and outcomes improved?. *Lancet Neurology*.

[4] Jia Q, Liu LP, Wang YJ (2010). Stroke in China. *Clinical and Experimental Pharmacology and Physiology*.

[5] Panagos PD (2008). The approach to optimizing stroke care. *American Journal of Emergency Medicine*.

[6] Wardlaw JM, Murray V, Berge E, Del Zoppo GJ (2009). Thrombolysis for acute ischaemic stroke. *Cochrane Database of Systematic Reviews*.

[7] Sze FKH, Yeung FF, Wong E, Lau J (2005). Does Danshen improve disability after acute ischaemic stroke?. *Acta Neurologica Scandinavica*.

[8] Wu B, Liu M, Liu H (2007). Meta-analysis of traditional Chinese patent medicine for ischemic stroke. *Stroke*.

[9] Ceniceros S, Brown GR (1998). Acupuncture: a review of its history, theories, and indications. *Southern Medical Journal*.

[10] Zhao XF, Du Y, Liu PG, Wang S (2012). Acupuncture for stroke: evidence of effectiveness, safety, and cost from systematic reviews. *Topics in Stroke Rehabilitation*.

[11] Kong JC, Lee MS, Shin BC, Song YS, Ernst E (2010). Acupuncture for functional recovery after stroke: a systematic review of sham-controlled randomized clinical trials. *Canadian Medical Association Journal*.

[12] Liu Z, Guan L, Wang Y, Xie CL, Lin XM, Zheng GQ (2012). History and mechanism fortreatment of intracerebral hemorrhage with scalp acupuncture. *Evidence-Based Complementary and Alternative Medicine*.

[13] WHO Scientific Group on International Acupuncture Nomenclature A Proposed Standard International Acupuncture Nomenclature.

[14] Lu SK (1991). Scalp acupuncture therapy and its clinical application. *Journal of Traditional Chinese Medicine*.

[15] Zheng GQ (2009). Methodological standards for experimental research on stroke using scalp acupuncture. *Acupuncture & Electro-Therapeutics Research*.

[16] Zheng GQ, Zhao ZM, Wang Y (2011). Meta-analysis of scalp acupuncture for acute hypertensive intracerebral hemorrhage. *Journal of Alternative and Complementary Medicine*.

[17] Hsing WT, Imamura M, Weaver K, Fregni F, Azevedo Neto RS (2012). Clinical effects of scalp electrical acupuncture in stroke: a sham-controlled randomized clinical trial. *Journal of Alternative and Complementary Medicine*.

[18] Jorgensen HS, Nakayama H, Raaschou HO, Vive-Larsen J, Stoier M, Olsen TS (1995). Outcome and time course of recovery in stroke. Part II: time course of recovery. The Copenhagen Stroke Study. *Archives of Physical Medicine and Rehabilitation*.

[19] Moher D, Liberati A, Tetzlaff J, Altman DG, PRISMA Group (2009). Preferred reporting items for systematic reviews and meta-analyses: the PRISMA statement. *PLOS Medicine*.

[20] Wang XD (1988). Diagnostic essentials of various cerebrovascular diseases. *Chinese Journal of Neurology*.

[21] Chen QT (1996). Classification, diagnostic criteria and evaluation of neurological impairment for stroke patients. *Chinese Journal of Neurology*.

[22] Tang QS, Sun ST (1996). Clinical and experimental research on acute cerebral infartion treated by needing Shu Xue on head. *Journal of Beijing College of Traditional Chinese Medicine*.

[23] Yu CD, Wu BH, Hong AH, Bei JY, Yu Z (2004). Changes of serum MDA content and neurological rehabil itation in cerebral infarction patients treated with scalp- acupuncture plus medication. *Acupuncture Research*.

[24] Wei TH, Xin TL, Tang Q (2005). The evaluation of scalp acupuncture combined with recovery techniques on the movement dysfunction after cerebral infarction. *Chinese Journal of Rehabilitation Theory and Practice*.

[25] Xu HB, Zhou GX, Li NP (2007). Treatment of acute cerebral infarction with scalp acupuncture combined with Yinxingdamo injection. *Journal of Clinical and Experimental Medicine*.

[26] Chen J, Jing L (2008). Clinical application of cluster needling of scalp point therapy combined with swallowing functional training in treatment of dysphagia after acute cerebral infarction. *Chinese Journal of Trauma and Disability Medicine*.

[27]  Yu CD, Wang GS, Wu BH, Li ZW, Song HM, Yu Ze (2010). Effects of cranial sutures acupuncture plus drugson recovery of neural function and serum SOD and MDA in acute cerebral infarction patients with phlegm syndrome. *Journal of Fujian University of TCM*.

[28] Zhang LR, Sa BY, Luo HL, Dong GR, Bao CL, Guo YQ (2011). Effect of bilateral scalp-acupoint penetrating acupuncture on serum levels of VEGF. *Chinese Journal of Rehabilitation*.

[29] Zhu GQ, Huang SY (2011). Effect of Xingnaoyinyang penetration needling method on the level of Ca^2+^ in serum of patients with acute cerebral infarction. *Journal of Emergency in Traditional Chinese Medicine*.

[30] Furlan AD, Pennick V, Bombardier C, van Tulder M (2009). 2009 Updated method guidelines for systematic reviews in the cochrane back review group. *Spine*.

[31] De Angelis C, Drazen JM, Frizelle FA (2004). International Committee of Medical Journal Editors. Clinical trial registration: a statement from the International Committee of Medical Journal Editors. *The New England Journal of Medicine*.

[32] Pildal J, Hróbjartsson A, Jörgensen KJ, Hilden J, Altman DG, Gøtzsche PC (2007). Impact of allocation concealment on conclusions drawn from meta-analyses of randomized trials. *International Journal of Epidemiology*.

[33] MacPherson H, Altman DG, Hammerschlag R (2010). Revised standards for reporting interventions in clinical trials of acupuncture (STRICTA): extending the consort statement. *Acupuncture in Medicine*.

[34] Duncan PW, Jorgensen HS, Wade DT (2000). Outcome measures in acute stroke trials: a systematic review and some recommendations to improve practice. *Stroke*.

[35] Vickers A, Goyal N, Harland R, Rees R (1998). Do certain countries produce only positive results? A systematic review of controlled trials. *Controlled Clinical Trials*.

[36] Zhang SH, Liu M, Asplund K, Li L (2005). Acupuncture for acute stroke. *Cochrane Database of Systematic Reviews*.

[37] (1998). NIH consensus conference. Acupuncture. *The Journal of the American Medical Association*.

[38] Zhang J, Shang H, Gao X, Ernst E (2010). Acupuncture-related adverse events: a systematic review of the chinese literature. *Bulletin of the World Health Organization*.

[39] Peuker E, Filler T (2004). Guidelines for case reports of adverse events related to acupuncture. *Acupuncture in Medicine*.

[40] Sharrack B, Hughes RAC, Soudain S, Dunn G (1999). The psychometric properties of clinical rating scales used in multiple sclerosis. *Brain*.

[41] Bian ZX, Chang YH (2011). Revised STRICTA as an extension of the CONSORT statement: more items should be involved in the checklist. *Journal of Alternative and Complementary Medicine*.

